# RESULTS OF A DOUBLE-BLIND RCT OF THE FUNCTIONAL EFFECT OF KETONE ESTERS IN OLDER ADULTS

**DOI:** 10.1093/geroni/igae098.3705

**Published:** 2024-12-31

**Authors:** Brianna Stubbs, Elizabeth Stephens, Chatura Senadheera, Sawyer Peralta, Stephanie Roa Diaz, Wendie Silverman-Martin, Laura Alexander, John Newman

**Affiliations:** Buck Institute, Novato, California, United States; Buck Institute for Research on Aging, Novato, California, United States; Buck Institute, Novato, California, United States; Buck Institute, Novato, California, United States; Buck Institute, Novato, California, United States; Buck Institute, Novato, California, United States; Buck Institute, Novato, California, United States; Buck Institute for Research on Aging, Novato, California, United States

## Abstract

**Background:**

Ketone bodies are metabolites produced during fasting or on a ketogenic diet that have pleiotropic effects on the inflammatory and metabolic aging pathways underpinning frailty in in vivo models. Ketone esters (KEs) are compounds that induce hyperketonemia without dietary changes and may impact physical and cognitive function in young adults. The functional effects of KEs have not been studied in older adults.

**Objectives:**

Our long-term goal is to examine if KEs modulate aging biology mechanisms and clinical outcomes relevant to frailty in older adults. Here, we report the exploratory functional and quality-of-life outcome measures collected during a 12-week safety and tolerability study of KE (NCT05585762). Design: Randomized, placebo-controlled, double-blinded, parallel-group, pilot trial of 12-weeks of daily KE ingestion. Setting: The Buck Institute for Research on Aging, California. Participants: Community-dwelling older adults (≥ 65 years), independent in activities of daily living, with no unstable medical conditions (n = 30). Intervention: Subjects were randomly allocated (1:1) to consume 25 g daily of either KE (bis-octanoyl (R)-1,3-butanediol) or a taste, appearance, and calorie-matched canola oil placebo (PLA). Measurements: Longitudinal change in physical function, cognitive function and quality of life were analyzed as exploratory outcomes in completers (n = 11 PLA, n = 12 KE). A composite vigor-frailty functional outcome was calculated. Heart rate and activity were followed using digital wearables.

**Results:**

There were no statistically significant longitudinal differences between groups in exploratory functional, activity-based or quality of life outcomes in this pilot study.

